# The Effectiveness and Safety of *Tripterygium wilfordii Hook. F* Extracts in Rheumatoid Arthritis: A Systematic Review and Meta-Analysis

**DOI:** 10.3389/fphar.2018.00356

**Published:** 2018-04-16

**Authors:** Ying-Yan Zhou, Xuan Xia, Wen-Ke Peng, Qin-He Wang, Jian-Hong Peng, Yan-lin Li, Jian-Xiong Wu, Jian-Yong Zhang, Yue Zhao, Xiu-Min Chen, Run-Yue Huang, Per-Johan Jakobsson, Ze-Huai Wen, Qing-Chun Huang

**Affiliations:** ^1^Key Unit of Methodology in Clinical Research, Department of Rheumatology, The Second Affiliated Hospital, Guangzhou University of Chinese Medicine, Guangdong Provincial Hospital of Chinese Medicine, Guangzhou, China; ^2^Postdoctoral Mobile Research Station, Guangzhou University of Chinese Medicine, Guangzhou, China; ^3^Guangzhou Panyu Sanatorium, Guangzhou, China; ^4^Department of Rheumatology, Chinese Medicine Hospital, Jieyang, China; ^5^Department of Rheumatology, Dongguan Hospital of Traditional Chinese Medicine, Dongguan, China; ^6^Department of Rheumatology, Zhongshan Hospital of Chinese Medicine, Zhongshan, China; ^7^Department of Rheumatology, Guangzhou Orthopedic Hospital, Guangzhou, China; ^8^Department of Rheumatology, Shenzhen Traditional Chinese Medicine Hospital, Shenzhen, China; ^9^Guangdong Provincial Key Laboratory of Clinical Research on Traditional Chinese Medicine Syndrome, Guangzhou, China; ^10^Rheumatology Unit, Department of Medicine, Solna, Karolinska Institutet, Stockholm, Sweden; ^11^Rheumatology Clinic, Karolinska University Hospital in Solna, Stockholm, Sweden

**Keywords:** *Tripterygium wilfordii Hook. F*, rheumatoid arthritis, efficacy, safety, meta-analysis

## Abstract

**Objective:** To conduct a meta-analysis of the effectiveness and safety of *Tripterygium wilfordii Hook. F* (TwHF) extracts for the treatment of rheumatoid arthritis (RA).

**Methods:** A systematic literature search was conducted in PubMed, EMBASE, Cochrane, Medline, CNKI, SinoMed and WanFang Library till 12 July 2017. All included studies were analyzed with the use of the Review Manager 5.2 software according to the Preferred Reporting Items for Systematic Reviews and Meta-Analyses (PRISMA) Statement protocol.

**Results:** Fourteen randomized controlled trials (RCTs) were identified. TwHF extracts provided a statistically significant improvement in grip strength (GS), swelling joint count (SJC) and morning stiffness (MS) compared with placebo (*P* < 0.001). The meta-analysis showed significant differences between TwHF extract-treated group and the DMARDs group in GS, MS, C-reactive protein (CRP), and tender joint count (TJC) (*P* < 0.05), aside from ESR and SJC (*P* > 0.05). The pooled results also displayed significant differences between the combination of TwHF extracts with DMARDs and the DMARDs alone group in ESR, CRP, SJC, and TJC (*P* ≤ 0.05). For the safety analysis, two trials favored TwHF extract-treatment and one trial favored non-TWHF extract-treatment in AEs (*P* < 0.05). Eleven trials showed no statistically significant differences between TwHF extract-treated group and the DMARDs group (*P* > 0.05).

**Conclusions:** The findings of this systematic review with meta-analysis indicate that TwHF extracts provides statistically significant and clinically important improvement in RA symptoms and has an acceptable safety profile.

## Introduction

Rheumatoid arthritis (RA) is an autoimmune disease of still unknown etiology that is characterized by systemic, destructive, and progressive inflammatory polyarthritis (Smolen et al., 2016). RA could lead to joint deformity, disability, and increased mortality if inadequately treated (Picerno et al., 2015). According to the guidelines proposed by the American College of Rheumatology (ACR) for the management of RA (Singh et al., 2016), disease-modifying anti-rheumatic drugs (DMARDs) and specific targeted therapies (including inhibitors of tumor necrosis factor (TNF) and other novel biological compounds) are recommended to interfere with the disease process in RA. However, the discontinue rate after 2 years with TNF blocker is around 40–60% due to side effects, development of anti-drug antibodies and lack of efficacy (Iannone et al., 2015; Arends et al., 2017; Favalli et al., 2017). Furthermore, biologics are unlikely to be of general benefit in the developing world because of the financial constraints (Hodkinson et al., 2014) although costs are decreasing as several original drugs are subject for competition with recent biosimilars *Tripterygium wilfordii Hook F* (TwHF) is a member of the Celastraceae family and is abundant in south China (Tao and Lipsky, 2000). Anti-inflammatory and immunosuppressive compounds extracted from TwHF have been used for the treatment of a wide spectrum of autoimmune and inflammatory diseases, including RA (Fan and Li, 2006; Zeng et al., 2009), ankylosing spondylitis (AS) (Li et al., 2016), and systemic lupus erythematosus (SLE) (Du et al., 2000). Additionally, TwHF extracts have been demonstrated to exert beneficial effects on nephrotic syndrome (Jiang, 1994), Crohn's disease (Ren et al., 2007), and solid tumors (Yang et al., 2003). The potential molecular mechanisms underlying the anti-inflammatory and immunosuppressive effects of TwHF extracts includes the inhibition of platelet activation (Hu et al., 2009), nitric oxide induction (Wang et al., 2004), as well as prostaglandin E2 production (Geng et al., 2012). Based on studies both *in vitro* and *in vivo*, it is reasonable to speculate that TwHF extracts represent herbal DMARDs, which is different from synthetic DMARDs. Importantly, TwHF extracts have been listed in the Clinical Guidelines for diagnosis and treatment of RA in China since 2004.

Only a few multicenter clinical trials have been performed to confirm the effects of TwHF extracts in the treatment of RA, except for the trial conducted by Peking Union Medical College Hospital in 2015 (Lv et al., 2015). The symbolic trial proved that TwHF monotherapy was not inferior to methotrexate (MTX) monotherapy, while the combination of MTX and TwHF was better than MTX monotherapy, with respect to effects in controlling disease activity in patients with active RA. Although several systematic reviews and meta-analyses regarding the efficacy and safety of TwHF extracts in the treatment of RA have been reported, these systematic reviews are dated and achieved, to some extent, the contradictory conclusions (Canter et al., 2006; Jiang et al., 2009; Cameron et al., 2011; Little and Parsons, 2011; Liu et al., 2013). This study intends to re-systematically review the potential effects and safety of the TwHF extracts in the treatment of RA with regard to the published articles recently.

### Methods

We conducted and reported this review according to the recommendations of the Preferred Reporting Items for Systematic Reviews and Meta-Analysis (PRISMA).

#### Search strategy for identification of studies

We searched the following digital databases to identify trials: PubMed, Embase, Medline and Cochrane. In addition, we searched the Chinese databases: CNKI Database, VIP Database, CBM Database and WanFang Database. All of the databases were searched to identify all relevant human clinical studies published until 12 July 2017. For the English databases, the search strategy used was as follows: ([“*Tripterygium wilfordii* Hook F”] OR [“*Tripterygium wilfordii*”] OR [“*Tripterygium*” or “thunder god vine”] OR [“lei gong teng”]) AND ([“rheumatoid arthritis”] OR [“RA”]) AND ([“random control trials”] OR [“RCT”]). For the Chinese databases, free text terms were used, such as “lei gong teng” (which means *Tripterygium wilfordii* Hook F in Chinese) and “lei feng shi guan jie yan” (which means rheumatoid arthritis in Chinese) and “sui ji dui zhao shi yan” (which means RCT in Chinese). To collect an adequate number of trials, the reference lists of relevant publications were also searched to identify additional studies.

#### Selection criteria

Studies were selected for subsequent analyses if they satisfied the following criteria: (1) the study was a RCT with a parallel or crossover design regardless of blinding; (2) people enrolled were diagnosed with RA, according to the 1987 guidelines of the American Rheumatology Association (Arnett et al., 1988), and were excluded with any other autoimmune diseases; (3) TwHF extracts were used as an active treatment intervention whether the subjects took extracts of TwHF alone or with other DMARDs for at least 4 weeks; (4) the outcomes included tender joint count (TJC), swelling joint count (SJC), grip strength (GS), morning stiffness (MS), erythrocyte sedimentation rate (ESR), C-reactive protein (CRP) and AEs.

#### Data extraction and management

Two examiners selected the articles and extracted the relevant data independently. Divergences were resolved by consensus. Based on the PRISMA requirements, a flow diagram of the study selection has been generated. Essential information from each trial was collected: study design, characteristics of participants, intervention and dosing regimen, concomitant therapy, duration of treatment, and clinical outcomes. All studies were also scored by two independent reviewers in accordance to the Cochrane Collaboration's Risk of Bias tool (Higgins and Green, 2014).

#### Statistical analysis

All included studies were analyzed with the use of the Review Manager 5.2 software.

For continuous outcomes, results were summarized using a mean difference and a 95% confidence interval (CI). Heterogeneity was evaluated statistically using the *I*^2^ statistic. The meta-analyses were carried out using a random effects model if *I*^2^ > 50% but a fixed effects model if *I*^2^ ≤ 50%. A significance level of 5% was used for all statistical tests.

### Results

#### Literature search results

**Figure 1:**
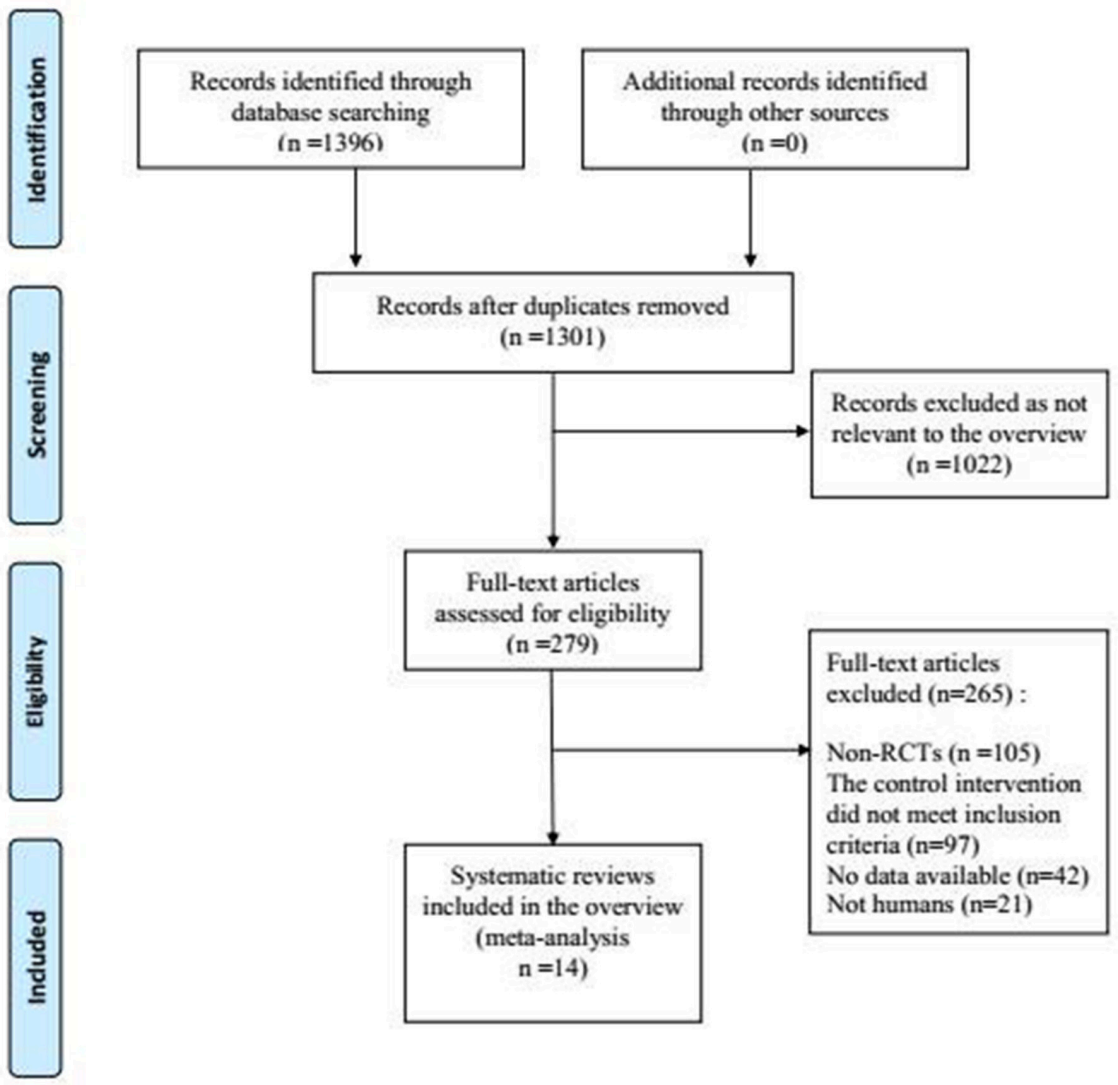
Process of searching and screening studies.

The process of study selection is shown in Figure 1. According to the selection criteria defined in the Methods section, 14 RCTs were included for systematic review and 14 RCTs were included in the meta-analysis. The characteristics of the included trials are shown in Table 1.

**Table 1:** The characteristics of the included trials.

**Author**	**sex ratio (M/F)**	**age(Y)**	**Number of patients**		**Intervention and dose**		**Duration**	**Outcomes**
			**Experimental**	**Control**	**Experimental**	**Control**		
Tao et al., 1989	N/A	N/A	27	31	TwHF 60 mg/d	PBO	12 W	SJC, MS, GS, ESR, AE
Cibere et al., 2003	63/27	40.5	31	30	TwHF(N/A)	PBO	6 W	TJC, SJC, MS,GS, ESR,CRP, AE
Liu et al., 2006	6/24	48	10	10	TwHF 30 mg, tid	MTX 7.5 mg, qw	3 M	TJC, SJC, MS, ESR, CRP, AE
Yang and Zhang, 2007	34/86	36.7	60	60	TwHF 20 mg, tid	MTX 15 mg, qw	1 M	TJC, SJC, MS,GS, ESR, CRP, AE
Goldbach-Mansky et al., 2009	61/60	53	60	61	TwHF 180 mg/d	SSZ 2 g/d	24 W	ESR, CRP, AE
Yang, 2011	13/133	54.2	74	72	TwHF (contains triptolide 198 μg/d)	MTX 10 mg, qw	3 M	TJC, SJC, MS,GS, ESR, CRP, AE
Lv et al., 2015	59/79	51.1	69	69	TwHF 20 mg,tid	MTX 7.5~12.5 mg, qw	12 W	TJC, SJC, ESR, CRP, AE
	62/76	50.8		69	TwHF 20 mg, tid + MTX 7.5~12.5 mg, qw	MTX 7.5~12.5 mg, qw	12 W	TJC, SJC, ESR, CRP, AE
Ou et al., 2016	23/27	45.5	25	25	TwHF 10 mg, qd + MTX 10 mg, qw	MTX 10 mg, qw	N/A	MS, ESR, CRP
Wang et al., 2017	34/62	55.5	48	48	TwHF 20 mg, tid + MTX 10 mg, qw	MTX 10 mg, qw	3 M	TJC, SJC, GS, ESR,CRP, AE
Cui and Yang, 2016	24/62	66.9	43	43	TwHF 20 mg, tid + LEF 20 mg, qd	LEF 10 mg, qd	6 M	TJC, SJC, MS, ESR, CRP
Li et al., 2017	15/45	42.3	30	30	TwHF 20 mg, tid + MTX 10 mg, qw + LEF 10 mg, qd	MTX 10mg, qw + LEF 10 mg, qd	12 W	ESR, CRP, AE
Wang et al., 2016	21/29	54.8	25	25	TwHF 20 mg, tid + MTX 10 mg, qw	MTX 10 mg, qw	3 M	TJC, SJC, ESR, CRP, AE
Feng and Ma, 2013	2/18	51.5	22	20	MTX 10 mg, qw+ TwHF 60 mg/d	MTX 10 mg, qw	3 M	TJC, SJC, ESR, CRP, AE
Chen et al., 2011	20/48	39	34	34	TwHF 20 mg, tid + MTX 10 mg, qw	LEF 20 mg, qn +MTX 10 mg, qw	12 W	TJC, SJC, ESR, CRP, AE

*TwHF, Tripterygium wilfordii Hook; TJC, tender joint count; SJC, swollen joint count; MS, morning stiffness; GS, grip strength; ESR, erythrocyte sedimentation rate; CRP, C-reactive protein; AE, adverse event; MTX, methotrexate; LEF, leflunomide; PBO, placebo*.

#### Quality of included systematic studies

**Figure 2:**
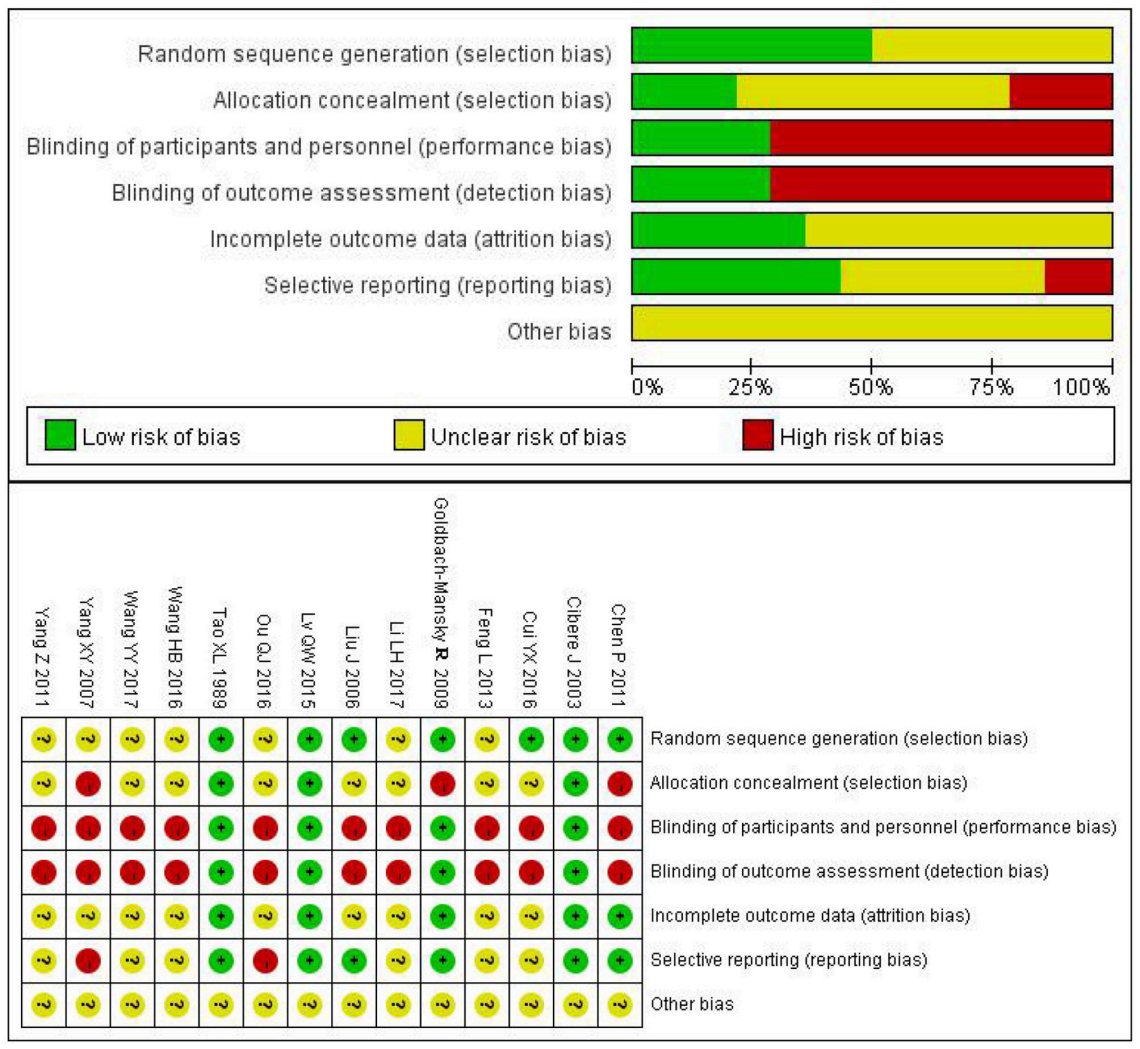
Risk of bias summary and risk of bias graph.

50% (7/14) of the studies mentioned a random design, and only 21% (3/14) described the blinded design; 29% (4/14) described the blinding of participants, personnel and outcome assessment; 36% (5/14) described complete outcome data; 14% (2/14) didn't report the AEs and thus were included in the studies of selective reporting (Figure 2).

#### Effects of interventions

**Figure 3:**
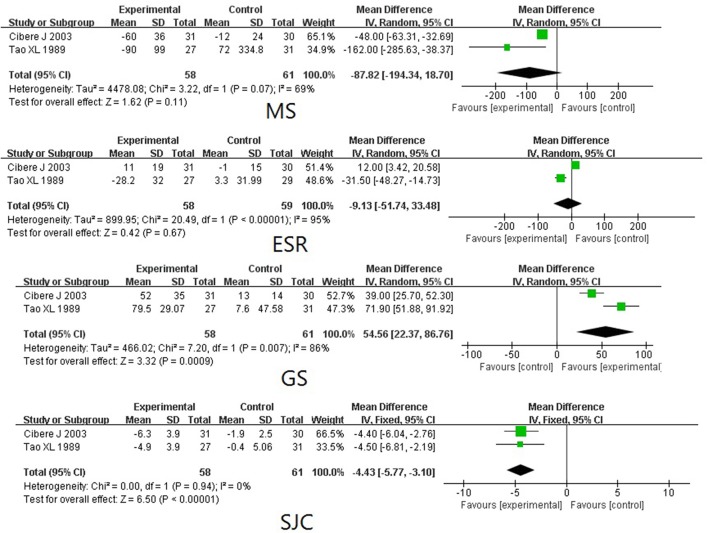
Forest plots of TwHF extracts treatment compared with a PBO. PBO, placebo; SJC, swollen joint count; MS, morning stiffness; GS, grip strength; ESR, erythrocyte sedimentation rate.

**Figure 4:**
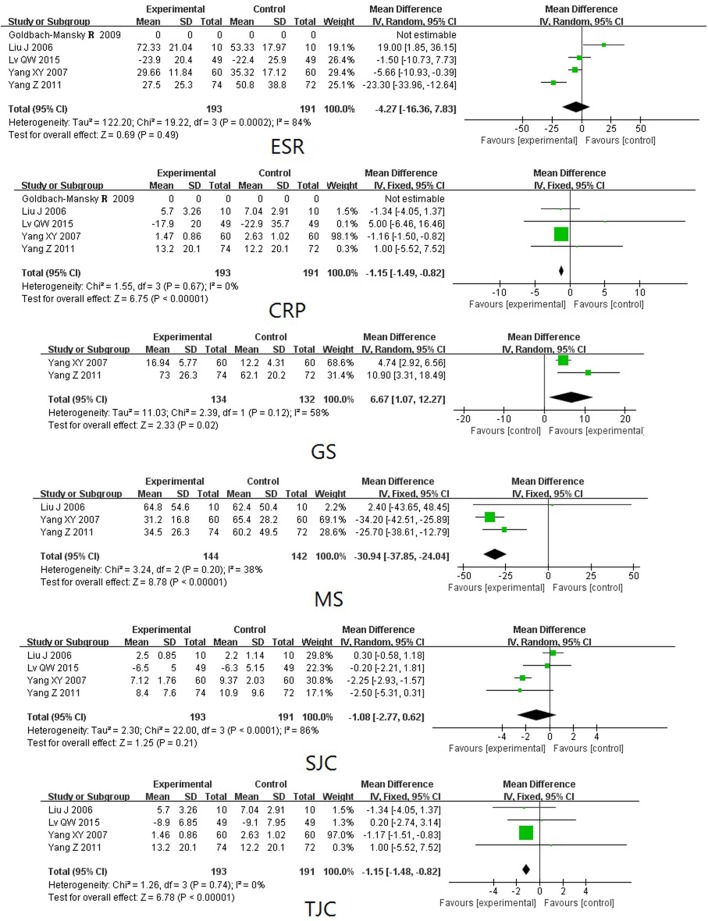
Forest plots comparing the effects of TwHF extracts treatment with DMARDs. DMARDs, disease-modifying antirheumatic drugs; TJC, tender joint count; SJC, swollen joint count; MS, morning stiffness; GS, grip strength; ESR, erythrocyte sedimentation rate; CRP, C-reactive protein.

**Figure 5:**
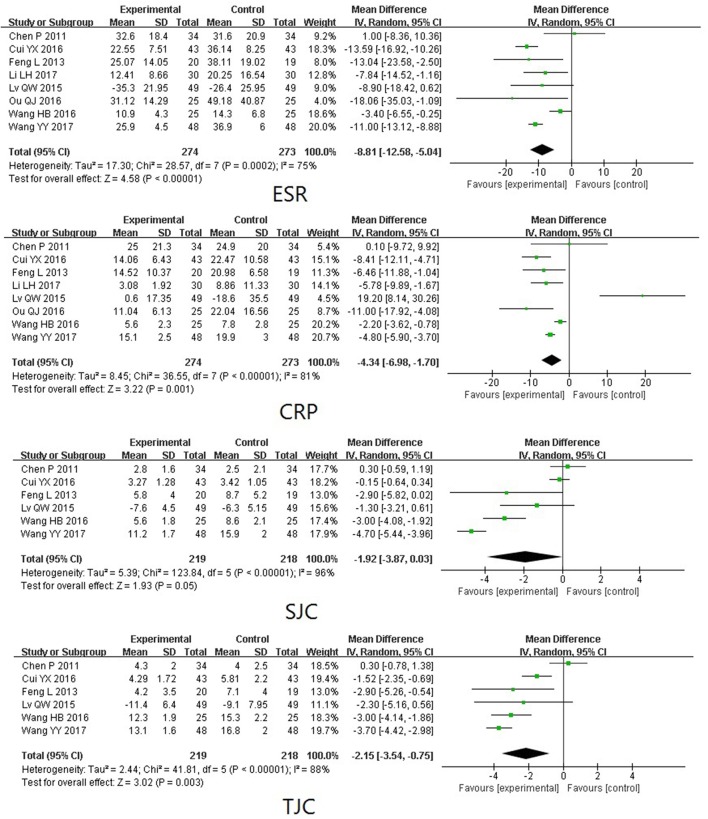
Forest plots comparing the effects of the co-administration of TwHF extracts and DMARDs with those of DMARDs Alone. DMARDs, disease-modifying antirheumatic drugs; TJC, tender joint count; SJC, swollen joint count; ESR, erythrocyte sedimentation rate; CRP, C-reactive protein.

1. TwHF extracts Compared with PBO
  Two trials (involving 119 patients) compared the therapeutic effects of TwHF extracts with PBO (Tao et al., 1989; Cibere et al., 2003). As shown in Figure 3, the pooled results indicated a significant difference between TwHF extracts-treated group and the PBO group in GS and SJC (*P* < 0.001), aside from MS and ESR (*P* > 0.05).
2. TwHF extracts Compared with DMARDs
  Four trials (involving 384 patients) compared the therapeutic effects of TwHF extracts with those of DMARDs (Liu et al., 2006; Yang and Zhang, 2007; Goldbach-Mansky et al., 2009; Yang, 2011). As illustrated in Figure 4, the pooled results displayed significant differences between TwHF extract-treated group and the DMARDs group, aside from ESR and SJC (*P* > 0.05).
3. The combination of TwHF extracts and DMARDs Compared with DMARDs Alone
  Eight trials (involving 547 patients) compared the therapeutic effects of “TwHF extracts +DMARDs” with DMARDs alone (Chen et al., 2011; Feng and Ma, 2013; Lv et al., 2015; Cui and Yang, 2016; Ou et al., 2016; Wang et al., 2016, 2017; Li et al., 2017). As illustrated in Figure 5, the pooled results displayed significant differences between the two groups in ESR, CRP, SJC, and TJC (*P* ≤ 0.05). Unfortunately, only one of the included trials reported GS and MS.
4. AEs
  Twelve trials reported outcomes for AEs. Seven trials (Yang and Zhang, 2007; Goldbach-Mansky et al., 2009; Chen et al., 2011; Yang, 2011; Wang et al., 2016, 2017; Li et al., 2017) reported mild to moderate gastrointestinal events in a few of the participants who received TwHF extracts. Menstruation disorders or amenorrhea was reported in 3 trials (Yang and Zhang, 2007; Goldbach-Mansky et al., 2009; Chen et al., 2011) in the TwHF extract group. Three trials (Goldbach-Mansky et al., 2009; Chen et al., 2011; Li et al., 2017) reported mild liver function abnormalities in a few patients caused by the intake of TWHF extracts. One trial (Li et al., 2017) reported skin rash in one patient caused by the intake of TWHF extracts. Two trials favored TwHF extract-treatment and one trial favored non-TWHF extract-treatment in AEs (*P* < 0.05). The other 11 trials showed no significant differences between the two groups.

## Discussion

The extracts of TwHF, including triptolide, tripdiolide, triptonide, and nearly 70 other constituents, have been shown to possess potent anti-inflammatory and immunosuppressive properties *in vitro* as well as in different animal models in numerous preclinical studies (Han et al., 2012). RA is a common autoimmune inflammatory polyarthritis. TwHF is approved in China and often used to treat a variety of immune and inflammation-related diseases, such as SLE and RA. The potential mechanism of TwHF extracts in treating RA is complicated. TwHF extracts can inhibit the expression of proinflammatory cytokines, proinflammatory mediators, adhesion molecules, and matrix metalloproteinases in macrophages, lymphocytes, synovial fibroblasts, and chondrocytes. It can also induce the apoptosis and inhibit the proliferation of lymphocytes and synovial fibroblasts (Bao and Dai, 2011).

In this study, we included 14 trials and set three subgroups to minimize the heterogeneity, along with four more new studies, including the recent multi-center trial with high quality (Lv et al., 2015), which made our systematic review different from the previous ones. The systematic reviews published by Cameron et al. (2011) (included 4 studies) and Canter et al. (2006) (included 2 studies) both indicated that TwHF extracts have beneficial effects in improving RA symptoms of RA but it may be associated with several side effects. The meta-analysis published by Jiang et al. (2009) included 7 trials with 393 participants. In this review, two subgroup analyses were performed. i.e., TwHF extracts vs. PBO and TwHF extracts vs. DMARDs. They found the efficacy of TwHF extracts for treatment of RA to be non-inferior to that of DMARDs on ESR, which is in accordance with the results of this article. However, as presented in the most recent review published by Liu et al. (2013), no beneficial effects on ESR and CRP were observed when the co-administration of TwHF extracts and DMARDs was compared with the administration of DMARDs alone. Our results support the findings by Jiang et al. and the explanation to the contradictory results by Liu et al. may originate from differences in the control groups used in the respective studies. Briefly, previous studies have revealed the potential value of TwHF extracts in treating RA, but they did not reach an agreement on the superiority of TwHF extracts to other drugs. Further multi-center, large-sample clinical trials are needed to evaluate the long-term effects of TwHF, including efficacy, safety, and tolerability.

The findings of this systematic review with meta-analysis indicate that TwHF extracts monotherapy or with background DMARDs provides statistically significant and clinically important improvement in RA symptoms and has an acceptable safety profile. The most common AEs with TwHF extracts were gastrointestinal discomfort, menstruation disorders or amenorrhea, and liver function damage, and they could be relieved with or without dose reductions. A previous study showed a dose-dependent efficacy but dose-independent safety of TwHF extracts in treating RA (Tao et al., 2002). The doses of TwHF extracts used in the 14 studies varied from 60 to 180 mg/day. Subgroup analysis of the AEs were not performed due to different interventions and the limited data. As mentioned above, TwHF extracts include triptolide, tripdiolide, triptonide and nearly 70 other constituents. Tripterygium Glycosides Tablets, used in all of the 14 enrolled studies, includes 6 kinds of components including triptolide, triptonide, tripterine, wilforgine, wilforine, and wilforlidea. The diversity of the components makes it difficult to analyze the safety of TwHF extracts. TwHF extracts may cause AEs by affecting lipid metabolism (Li et al., 2011), peroxisome proliferator-activated receptor signaling pathways, and cellular stress, as well as through inhibition of the liver mitochondrial respiratory chain (Fu et al., 2011). Although TwHF extracts showed an acceptable safety profile compared to DMARDs in this article, there is a clear need for improved understanding of contributing risk factors, as well as of prevention and management strategies to improve patients' tolerance for TwHF extracts.

Some limitations of this meta-analysis should be noted. First, due to the limitation of the searching condition, only the PubMed, Embase, Medline, Cochrane, CNKI, VIP, CBM, and WanFang databases were included in this study. Second, only 14 trials met the inclusion criteria and 85.7% (12/14) were conducted in China. Third, the included studies generally had major methodology flaws, such as inadequate randomization, double-blinding, and allocation concealment. Fourth, for included studies that had higher heterogeneity, only the random effect model was available in most cases, which may have certain confounding effects on the results. Fifth, differences in the doses of TwHF extracts and durations of treatment in the 14 studies may be contributable to the heterogeneity, while subgroup analysis were not performed in this study due to limitation of the small sample. Therefore, it need more strong evidence to confirm the effects of TwHF extracts on management of RA.

## Conclusions

Our findings suggest that TwHF extracts provides statistically significant and clinically important improvement in RA symptoms and has an acceptable safety profile. TwHF extracts could be considered a suitable treatment option for RA, especially to patients with contraindications to other therapies. Because there is a lack of long-term trials or reports of open studies running for more than 1 year, it is difficult to judge the full potential of TwHF for use in RA treatment. Further multi-center, large-sample clinical trials are needed to evaluate the long-term effects of TwHF, including efficacy, safety, and tolerability. The scientific evidence verifying that TwHF extracts are as effective as other conventional treatments in treating RA remains to be further validated.

## Author contributions

Y-YZ, XX, and R-YH contributed to the literature database search, data collection, data extraction, data analysis, and writing of the manuscript. W-KP, Q-HW, J-HP, Y-IL, J-XW, J-YZ, YZ, and X-MC performed data analysis and rationalization of the results. The topic was conceptualized by Z-HW, Q-CH, and P-JJ.

### Conflict of interest statement

The authors declare that the research was conducted in the absence of any commercial or financial relationships that could be construed as a potential conflict of interest. The reviewer SL and handling Editor declared their shared affiliation.
